# Near-Miss Diagnoses of Solitary Bladder Tumors Highlight the Importance of Immunohistochemical Staining

**DOI:** 10.1155/2020/8855451

**Published:** 2020-12-16

**Authors:** Van Schloegel, Syed M. Alam, Katie Dennis, Jeffrey Holzbeierlein, John A. Taylor III

**Affiliations:** Department of Urology, University of Kansas Health System, 3901 Rainbow Boulevard, Kansas City, KS 66160, USA

## Abstract

We report three cases of prostate adenocarcinoma appearing as bladder masses and misdiagnosed as muscle-invasive bladder cancer (MIBC). Patients were referred for consideration for radical cystectomy after initial pathological diagnosis suggested poorly differentiated bladder cancer. Pathological review of tissue samples and subsequent immunohistochemical (IHC) staining confirmed advanced prostatic adenocarcinoma. Systemic therapy for prostate cancer was then initiated. These cases highlight the importance of patient history, physical exam, and IHC staining in consideration of a bladder mass, as these patients may have been subject to undue morbidity and surgical intervention without accurate pathologic diagnosis.

## 1. Introduction

In 2020, prostate cancer and bladder cancer are expected to be diagnosed in 191,930 and 81,400 new patients, respectively [1]. Standard of care for a new bladder mass includes transurethral resection of bladder tumor (TURBT) for treatment, diagnosis, and initial staging. TURBT specimens appearing as poorly differentiated carcinomas, particularly from trigonal tumors, may be misdiagnosed as urothelial in origin without clinical suspicion and further evaluation. It is uncommon for invasive prostate cancer to present away from the trigone, and these tumors may be at even higher risk for misdiagnosis. This may lead to undue morbidity and mortality associated with systemic therapy and surgery for presumed bladder cancer. We describe three cases of near-miss diagnoses that emphasize the importance of appropriate IHC staining based on clinical suspicion and patient history.

## 2. Case Presentations

### 2.1. Case 1

**Figure 1:**
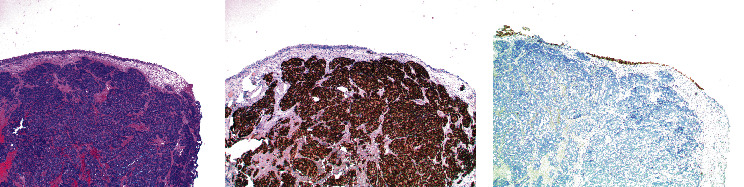
Negative GATA3 and positive NKX3.1 staining confirms prostatic adenocarcinoma. Low power view of the bladder tumor in (a) shows a normal urothelial mucosa and tumor beneath the urothelium, involving the submucosa. Underlying invasion of the muscularis propria was also present. (b) Strong diffuse nuclear staining with the immunohistochemical stain NKX3.1 was seen. Note the surface urothelium is negative. (c) The tumor cells are negative for the immunohistochemical stain GATA3, while the surface benign urothelium shows positive nuclear staining.

An 83-year-old male presented with an enhancing posterior wall bladder mass identified on surveillance imaging for a known renal mass. A 1.5 cm enhancing posterior peripheral zone prostate nodule was also noted. Cystoscopy revealed a nodular tumor in the posterior bladder wall with diffuse erythematous papillary changes along the right lateral wall. TURBT was performed with initial pathology reported as poorly differentiated bladder cancer. Of note, this patient had a history of localized prostate cancer previously treated with radiotherapy at a separate institute eight years prior. Prostate-specific antigen (PSA) was 2.57 ng/mL just prior to TURBT, with a PSA nadir of 0.2 ng/mL approximately three years prior. As such, additional IHC staining for GATA3 and NKX3.1 was performed on the TURBT specimen. This revealed diffuse strong nuclear staining of NKX3.1 with GATA3 positivity only seen in benign surface epithelium supporting a diagnosis of prostatic adenocarcinoma (Figure 1). Appropriate therapy for prostate cancer was subsequently initiated.

### 2.2. Case 2

**Figure 2:**
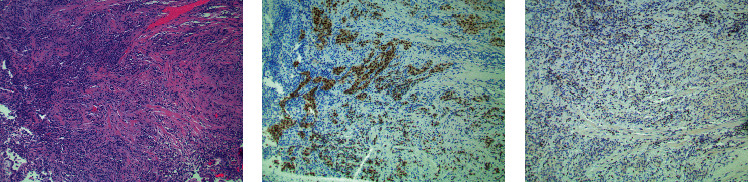
NKX3.1 distinguishes prostatic origin amidst significant cautery artifact. (a) Tumor cells can be seen infiltrating through smooth muscle tissue, which appear epithelioid, but otherwise show no distinguishing features. These tumor cells are in a background of inflammation and appear cauterized, making it difficult to classify based on morphology alone. (b) The immunohistochemical stain NKX3.1 shows strong nuclear positivity in the tumor cells. In contrast (c), the larger tumor cell nuclei are negative for the immunohistochemical stain GATA3 and only show nonspecific faint blushing of the cytoplasm. GATA3 is appropriately positive in an occasional small background of benign lymphocyte nuclei.

An 80-year-old male presented to his local urologist for worsening obstructive voiding symptoms. Flexible cystoscopy revealed a dense bladder neck contracture. The patient underwent transurethral incision of this contracture at which time abnormal papillary tissue was seen at the bladder neck. Transurethral resection of this tissue was reported as muscle-invasive urothelial carcinoma with squamous differentiation, prompting referral for radical cystectomy at our institution. The patient was counseled on surgery for MIBC but on physical exam was found to have a nodular prostate. As such, the patient was scheduled for repeat TURBT. PSA was drawn and measured 179.91 ng/mL, prompting internal pathological review. Initial tissue samples revealed poorly differentiated tumor cells with muscular invasion. Staining for NKX3.1 and GATA3 showed strong nuclear positivity in the tumor cells while GATA3 was appropriately confined to benign lymphocyte nuclei, confirming a diagnosis of prostate adenocarcinoma (Figure 2). TURBT was deferred, and staging studies revealed multiple sclerotic osseous lesions consistent with metastatic disease. Appropriate treatment for metastatic prostate cancer was initiated.

### 2.3. Case 3

A 79-year-old male underwent TURBT at an outside institution for a sessile lesion located at the bladder neck. Initial pathological review suggested a diagnosis of muscle-invasive urothelial carcinoma. As such, he was referred to our institution for consideration for radical cystectomy. The patient was noted to have a history of prostate adenocarcinoma previously treated with external beam radiation therapy. Digital rectal exam was unremarkable, and he did not have evidence of biochemical recurrence up to that time. Given this history and the location of his tumor, IHC staining for prostate cancer markers was pursued and revealed poorly differentiated prostate cancer. After further counseling, he went on to receive androgen deprivation therapy.

## 3. Discussion

In any patient with a bladder mass, a biopsy is needed to determine the grade, stage, and tissue origin. In patients previously treated with radiation therapy for prostate cancer, the tissue can appear as poorly differentiated carcinoma making organ specific cancer diagnosis challenging. The location of the mass may correlate with tissue of origin, although this is not always the case, as in our patient with tumors distinctly away from the trigone/bladder neck. Here, we demonstrate the importance of appropriate IHC staining in bladder tumor samples to differentiate urothelial malignancy from invasive prostate adenocarcinoma.

A retrospective analysis by Liu et al. demonstrated that up to 3.1% of all prostate cancers may originally be misdiagnosed as bladder cancer, although the sample size in this study was small [2]. Misdiagnosed tumors in this report appeared on imaging as masses along the bladder neck and trigone. There is little data available on the locations outside of these regions of the bladder that may harbor prostate cancer. In a recent meta-analysis, Zhao et al. demonstrated that the hazard ratio for the development of bladder cancer after radiation for prostate cancer is approximately 1.6 [3]. In the setting of prior radiotherapy for prostate cancer, a high clinical suspicion for urothelial origin of bladder tumors may be warranted, but progression of the primary prostate cancer cannot be excluded.

Appropriate staining for prostate adenocarcinoma in these scenarios should include a panel of prostatic and urothelial markers, including PSA, NKX3.1, and GATA3. In a study by Mohanty et al. looking at high-grade prostate cancer of the bladder neck, PSA showed a sensitivity of only 25%, while NKX3.1 had a sensitivity of 100%. In this same study, both PSA and NKX3.1 showed 100% specificity with no false positives in urothelial carcinomas [4]. GATA3 has been shown to have a sensitivity of 80% as a urothelial marker in high-grade urothelial carcinoma and did not stain positive in any of the 38 high-grade prostatic adenocarcinoma samples in a study by Chang et al. [5]. The decision to pursue additional staining in the appropriate clinical context is key to avoiding the undue harm associated with misdiagnosis.

## 4. Conclusion

These cases emphasize the importance of a thorough history and physical exam in patients presenting with a new bladder mass. They also highlight the value of appropriate IHC staining of bladder tumor samples to distinguish prostatic and urothelial origin of malignancies in select patients, regardless of tumor location.

## Abbreviations

MIBC: Muscle-invasive bladder cancer
TURBT: Transurethral resection of bladder tumor
PSA: Prostate-specific antigen
IHC: Immunohistochemical.
